# Refeeding Syndrome and Non-Alcoholic Wernicke’s Encephalopathy in a Middle-aged Male Initially Presenting with Gallstone Pancreatitis: A Clinical Challenge

**DOI:** 10.7759/cureus.5156

**Published:** 2019-07-17

**Authors:** Ifrah Butt, Nicolas Ulloa, Balarama K Surapaneni, Franklin Kasmin

**Affiliations:** 1 Internal Medicine, Graduate Medical Education, Aventura Hospital, Aventura, USA; 2 Gastroenterology, Aventura Hospital, Aventura, USA

**Keywords:** wernicke's encephalopathy, gallstone, recurrent pancreatitis, refeeding syndrome, pancreatic necrosis, pancreatitis

## Abstract

Wernicke’s Encephalopathy (WE) is a neurological condition characterized by ophthalmoplegia, ataxic gait, and altered mental status. It is an underdiagnosed yet severely limiting disease process precipitated by thiamine deficiency. Often times, it can occur in conjunction with other disease states like refeeding syndrome in which the underlying etiology is prolonged periods of malnutrition. We present a unique case of non-alcoholic WE in a middle-aged male who initially presented with gallstone pancreatitis complicated with severe metabolic derangements. This ultimately resulted in the development of non-alcoholic WE. Prevention of this condition is a clinical challenge for most physicians as the classic features associated with thiamine deficiency lack diagnostic sensitivity and specificity in critically ill patients. As a result, early recognition and prompt management of this can dramatically decrease morbidity and mortality. Our case highlights and emphasizes the importance of maintaining a high index of suspicion for WE and refeeding syndrome in the setting of altered sensorium and metabolic derangements.

## Introduction

Wernicke's encephalopathy (WE) is precipitated by thiamine deficiency and is more commonly seen in alcoholics [1]; however, it can also be seen in patients with malnutrition. Refeeding syndrome is a life-threatening derangement in fluid and electrolyte balance that is commonly encountered in the poorly nourished population. In the developed world, malnourishment is not as pervasive, which may lead to underdiagnosis of the syndrome due to unfamiliarity. The syndrome is commonly seen in patients receiving alternate means of nutrition as they most likely have conditions that predispose them to malnourishment [2]. The most dangerous complications involve cardiac failure, life-threatening arrhythmias, seizures, encephalopathy and anemia [3]. Our case demonstrates a rare clinical presentation of non-alcoholic WE that was precipitated by refeeding syndrome in the setting of prolonged bouts of biliary disease and associated complications.

## Case presentation

A 42-year-old African American male with a past medical history of hypertension and hyperlipidemia presented with severe, sharp, right upper quadrant abdominal pain associated with nausea and multiple episodes of non-bloody, non-bilious emesis. His physical examination was significant for right upper quadrant abdominal tenderness with positive murphy’s sign. Pertinent labs included elevated lipase of 2558 U/L, elevated amylase of 711 U/L, leukocytosis of 28.0 x 109/L and mild transaminitis. A computed tomography (CT) scan of the abdomen demonstrated acute pancreatitis, cholelithiasis, and pneumobilia. The patient was diagnosed with acute pancreatitis due to gallstones. Additionally, there was a high index of suspicion of incipient ascending cholangitis and gangrenous cholecystitis given the air in the biliary tree seen on abdominal imaging. He was admitted to the intensive care unit where he was stabilized with antibiotics and aggressive fluid resuscitation. Abdominal ultrasound demonstrated a non-dilated bile duct without evidence of stones. At this time, a decision was made to withhold feeds due to the severity of the patient’s symptoms further complicated by ileus. Over the course of his hospitalization, became septic and underwent placement of a percutaneous cholecystostomy tube. Culture of the biliary fluid was positive for Clostridium perfringens. With the antibiotics and placement of a biliary drain, the patient began to improve clinically. The plan was to continue him on oral antibiotics and discharge him home with the percutaneous cholecystostomy drain in place for 4-6 weeks until he was stable enough to undergo a cholecystectomy.

Approximately one month later, he was readmitted for persistent nausea and intractable vomiting. CT scan of the abdomen revealed interval development of a large fluid collection in the body and tail of the pancreas. He underwent endoscopic retrograde cholangiopancreatography (ERCP) with successful sphincterotomy, decompression of the biliary tree and pancreatic stent placement. A few days later, he also underwent placement of a transgastric stent from the pancreatic fluid collection to the stomach to allow for drainage of the large pancreatic phlegmon. The patient's condition improved and he was discharged when he was able to tolerate a clear liquid diet until his next visit. 

However, a few weeks later he was readmitted for the third time with persistent nausea, intractable vomiting and inability to tolerate any oral intake. A CT of the abdomen was repeated which showed persistent fluid in the pancreas measuring 3.6 x 2.5 cm and he underwent another endoscopic necrosectomy and he was discharged after a course of IV antibiotics when he was tolerating a clear liquid diet. A percutaneous endoscopic jejunostomy (PEJ) tube was considered but his family decided to defer the procedure.

A week later, he presented with his sister with episodes of confusion with waxing and waning forgetfulness. On physical exam, the patient was alert and oriented to person, place, and time. CT scan of the brain was unremarkable. It was unclear at this time what could have caused him to have changes in his mental status, but he appeared stable. Another CT scan of the abdomen was done which showed interval improvement of pancreatitis with a decrease in size of pseudocyst with drains and stents in place appropriately. This was deemed to be an appropriate window of time to perform PEJ tube placement. However, during the course of his hospital stay, he was noted to have waxing and waning mental status and progressively worsening lethargy which delayed the procedure further. Multiple CT scans of the brain were done which all were inconclusive. Due to his labile condition and high-risk hemodynamic instability, the PEJ placement was canceled and he was instead started on total parenteral nutrition (TPN) administered through a central line catheter. Of note, he had a weight loss of 30 kg within the last three months while being treated for his condition. 

**Figure 1:**
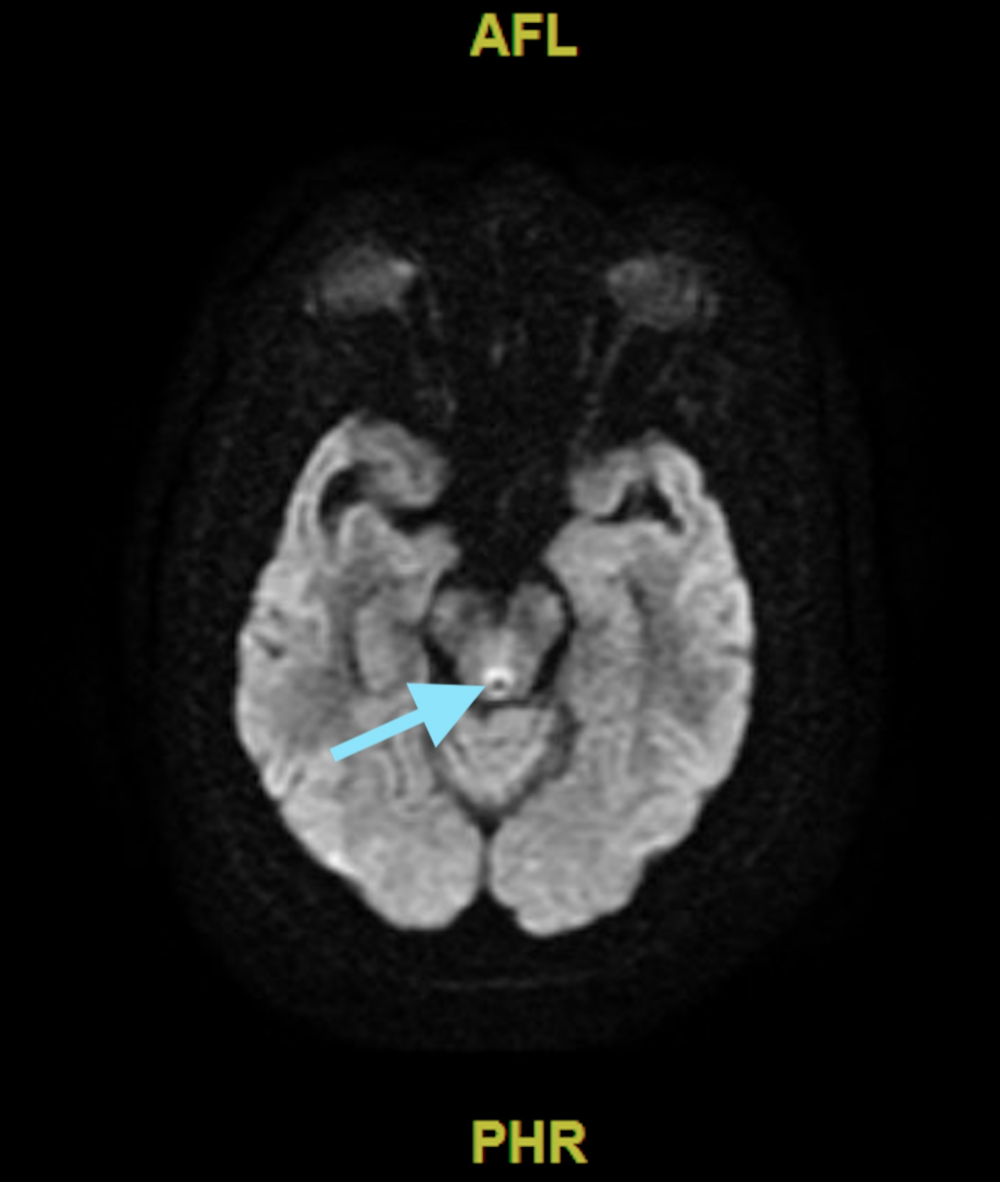
Magnetic e resonance imaging of the brain (diffusion-weighted) in axial section showing hyperintensity in the periaqueductal gray area depicted by the blue arrow.

**Figure 2:**
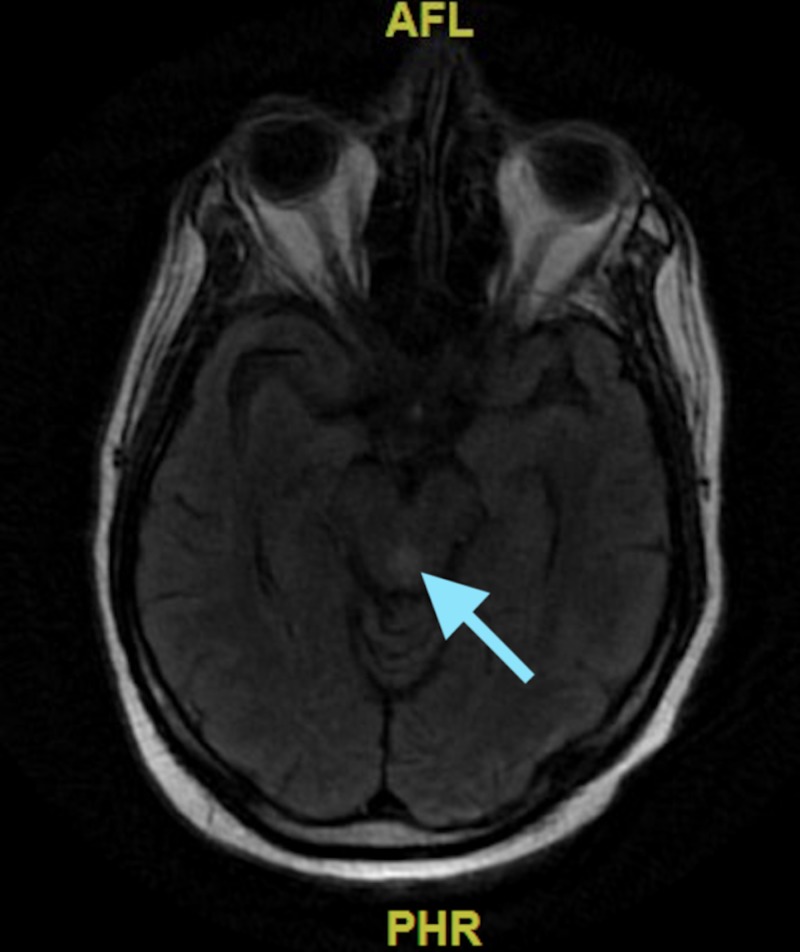
Magnetic resonance imaging of the brain (T2/FLAIR) showing hyperintensity in periaqueductal gray area depicted by the blue arrow.

Over the next few days, his cognitive function rapidly began to decline--lethargy worsened, he became very confused (only oriented to himself). His neurological exam was limited as he was only intermittently following commands and also developed ophthalmoplegia. Neurology was consulted and a magnetic resonance imaging (MRI) of the brain was performed which revealed increased signal on the diffusion-weighted (Figure 1) and T2/FLAIR images in the periaqueductal area (Figure 2). He was suspected to have non-alcoholics WE and was started on thiamine replacement therapy.

He was given thiamine 500 mg intravenously three times daily for three days, followed by 250 mg intravenously daily for a minimum of five days.

After three days of high dose intravenous thiamine, our patient showed significant improvement in his mental status. Although the improvement was gradual, it was clear that he was responding to the thiamine replacement therapy and eventually, his ophthalmoplegia improved as well.

## Discussion

Wernicke’s encephalopathy

Wernicke’s encephalopathy (WE) is a neurological disorder characterized by a nutritional deficiency in thiamine (vitamin B1) [4]. Although commonly seen in alcoholics, WE can be seen in any circumstance where nutritional demands are not being met, such as bariatric surgery, chronic diarrhea, malabsorption, cancer, anorexia nervosa, pancreatitis, refeeding syndrome, and chronic vomiting. It takes approximately 2-3 weeks of poor nutrition to develop nutritional deficiencies consistent with WE [1].

Thiamine is a coenzyme essential for various enzymes in the Kreb cycle and pentose phosphate pathway resulting in energy production within muscle, hepatocytes, and neurons. Deficiency leads to the buildup of lactic acid and reactive oxygen species [5]. This leads to neuronal cell death in the brain and most commonly affected areas include medial thalami, mammillary bodies, tectal plate, periaqueductal area of midbrain and periventricular regions of the third ventricle; atypical findings include regions of the cerebral cortex, cerebellum, and cranial nerve nuclei [4]. 

The classic triad of WE includes confusion, ophthalmoplegia, and ataxia [6]. In our patient's case, he had been experiencing chronic vomiting for 2-3 months secondary to severe complications of gallstone pancreatitis. Approximately 2 weeks prior to his admission, his wife noticed a decline in his mental status. Prolonged malnutrition in conjunction with disorientation and depressed levels of consciousness may be the predominating symptoms of WE and should be assessed in patients for early diagnosis and prompt initiation of thiamine repletion [7]. While admitted, our patient had waxing and waning mental status and ophthalmoplegia on physical exam. Alcoholic patients with WE are less likely to have ocular signs when compared to non-alcoholics with WE in a multicenter observational study [8]. Our patient was bed bound during most of his stay, but during sessions with physical and occupational therapy, he reported dizziness and inability to walk.

Although MRI and thiamine levels are useful in determining the diagnosis, non-alcoholic WE is a clinical diagnosis and should be suspected in any patient with a history of prolonged malnutrition. Chronic or recurrent vomiting can be an early symptom of thiamine deficiency and should be assessed for early diagnosis of thiamine deficiency and prevention of WE [6]. In this case, our patient had multiple risk factors including unintentional weight loss more than 15% in the past three to six months, little or no nutritional intake for more than 10 days, low levels of potassium, phosphate, or magnesium due to his poor oral intake as a result of his underlying condition of gallstone pancreatitis and history of chronic vomiting.

Refeeding syndrome

Refeeding syndrome (RFS) is a condition in which various metabolic changes are seen in patients with severe malnutrition upon initiation of nutrition. If excess calories are consumed too quickly, the body synthesizes glycogen and metabolizes glucose in an attempt to refill carbohydrate stores and produce adenosine triphosphate (ATP) at the cost of certain electrolytes, specifically phosphate, potassium and magnesium [2].

In terms of carbohydrate metabolism, phosphate is consumed in order to produce ATP and energy. Therefore, in RFS, excessive amounts of phosphate are spent in order to provide energy for a body recovering from starvation, which is why hypophosphatemia is seen in nearly 100% of RFS cases [1]. When our patient was admitted, his initial phosphate levels were critically low, consistent with RFS. Certain acute changes in phosphate can lead to hemolysis, rhabdomyolysis, cardiopulmonary changes and even death [1].

In addition to electrolyte imbalances, thiamine can also be affected during RFS. Thiamine is a coenzyme which is required throughout the process of glycolysis and can be acutely depleted as the body attempts to over-utilize glucose in RFS [3]. Therefore, symptoms consistent with Wernicke’s encephalopathy can be observed in addition to the metabolic derangements and changes consistent with hypophosphatemia, hypokalemia, and hypomagnesemia.

Guidelines from the National Institute for Health and Clinical Excellence recommend utilizing these criteria for identifying patients at high risk of refeeding problems [9]:

If the patient has one of the following: Body mass index (kg/m2) <16, unintentional weight loss >15% in the past three to six months, little or no nutritional intake for >10 days, low levels of potassium, phosphate, or magnesium before feeding.

Or if the patient has two or more of the following: Body mass index <18.5, unintentional weight loss >10% in the past three to six months, little or no nutritional intake for >5 days, history of alcohol misuse or drugs, including insulin, chemotherapy, antacids, or diuretics.

Patients at risk of RFS should have their phosphate, potassium, magnesium, and calcium levels monitored. Thiamine should be repleted with 200-300 mg daily PO to prevent RFS. Then the patient should be started on 0.0418 MJ/kg/day while slowly increasing feedings over 4-7 days. For severely malnourished patients, refeeding should be initiated at 0.021 MJ/kg/day [2].

## Conclusions

We presented a case of non-alcoholic WE in a patient who was diagnosed with gallstone pancreatitis. Our case highlights the importance of broadening your differential when presented with an array of symptoms that may appear benign in etiology. It becomes increasingly difficult to make a diagnosis when the clinical features associated with thiamine deficiency lack sensitivity and specificity in the critically ill population. Hence, MRI findings and thiamine levels aid in supporting the diagnosis. We hope our case cautions clinicians to suspect thiamine deficiency in patients with chronic recurrent vomiting and poor nutritional status in the setting of another condition such as gallstone pancreatitis. 
